# Medication-Related Osteonecrosis of Jaws: A Low-Level Laser Therapy and Antimicrobial Photodynamic Therapy Case Approach

**DOI:** 10.1155/2016/6267406

**Published:** 2016-09-07

**Authors:** Mariana Comparotto Minamisako, Guilherme Henrique Ribeiro, Mariáh Luz Lisboa, Mabel Mariela Rodríguez Cordeiro, Liliane Janete Grando

**Affiliations:** ^1^Dentistry Department, Oncology Research Center, Florianópolis, SC, Brazil; ^2^Postgraduate Program in Dentistry, Federal University of Santa Catarina, Florianópolis, SC, Brazil; ^3^Department of Morphological Sciences, Federal University of Santa Catarina, Florianópolis, SC, Brazil; ^4^Department of Pathology, Ambulatory of Stomatology, University Hospital, Federal University of Santa Catarina, Florianópolis, SC, Brazil

## Abstract

Medication-related osteonecrosis of the jaws (MRONJ) can be considered an inability of the alveolar bone to respond to an injury, which frequently leads to severe local and systemic complications. Once the problem is installed, dentist must use all therapeutic approaches recommended. This manuscript reports a successful management of MRONJ handled with antibiotics, conservative debridement, low-level laser therapy (LLLT), and photodynamic therapy (PDT) up to 12 months. As healing of MRONJ may be very slow, combined therapeutic approaches are required. Besides the recommended conventional treatment protocol, LLLT and PDT are important tools to contribute to healing and improvement of patient's quality of life.

## 1. Introduction

Medication-related osteonecrosis of the jaws (MRONJ) is considered an inability of alveolar bone to respond to injury. For example, when a tooth is extracted, there is a deep wound in the bone that is contaminated by oral microorganisms. Usually, there is local immune reaction and the bone promptly reacts to repair the wound. Macrophages and other inflammatory cells combat the bacterial contamination, osteoclasts remove any damaged bone, osteoblasts form new bone, and the epithelium regrows over the wound [1].

Antiresorptive and antiangiogenic agents are the known drugs in the etiology of MRONJ [2]. Bisphosphonates (BP) are the main antiresorptive medications that act with a great effect on alveolar bone, setting an imbalance between deposition (osteoblastic activity) and resorption (osteoclastic activity) [3].

MRONJ involves necrotic, exposed bone in the jaws, discomfort, possible secondary infection, swelling, painful mucosal lesions, and various dysesthesias [4]. Treatment of MRONJ should target to eliminate pain, control infection of hard and soft tissue, and minimize progression or occurrence of bone necrosis [5].

In recent years, low-level laser therapy (LLLT) has been employed as adjuvant therapy for treating MRONJ, since it presented great results in analgesia, ability to reduce edema formation and cell biomodulation, accelerating wound healing process [6]. On the other hand, photodynamic therapy (PDT) is recommended when infection and/or suppuration is present [7, 8]. Thus, the present case control study aimed to evaluate the effectiveness of LLLT and PDT in the management of MRONJ.

## 2. Case Report

**Figure 1:**
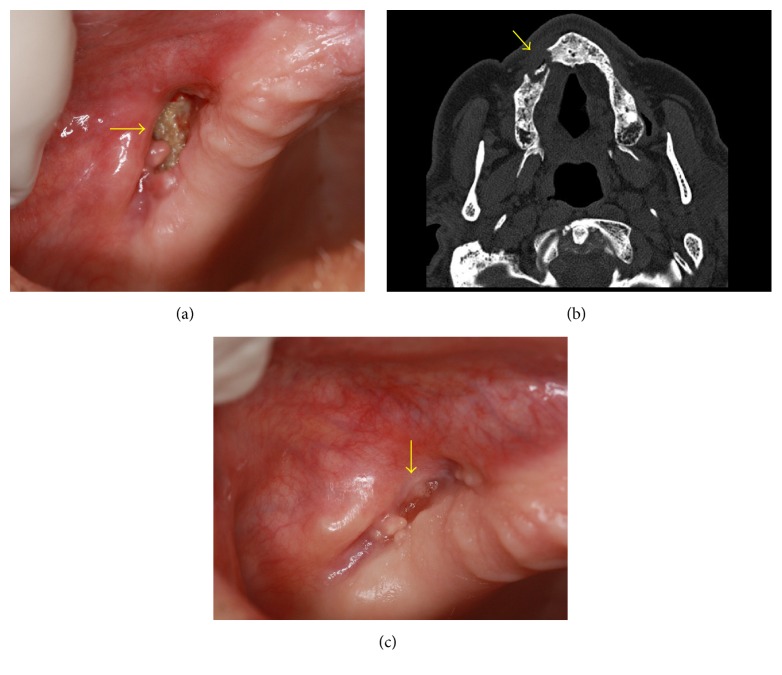
Clinical aspect of bone exposure measuring approximately 1.5 cm at vestibular sulcus of right maxilla (a). CT showing bone lysis and necrotic bone sequestrum at maxilla with oral antral communication risk (b). Clinical aspect of vestibular sulcus mucosa totally recovered after 37 sessions of LLLT and PDT (c).

A male 85-year-old patient was referred to the Stomatology Clinics with bone exposure, measuring approximately 1.5 cm at vestibular sulcus of right maxilla (Figure 1(a)), suppuration, pain, and putrescent smell. His medical background included weekly oral BP (alendronic acid 70 mg/week, for 8 years), due to bone thinning of both knees. Patient had no history of head and neck radiotherapy.

Clinical diagnosis of MRONJ was confirmed following the analysis of computed tomography (CT) images (Figure 1(b)). CT showed osteolysis and necrotic bone sequestrum formation at right maxilla with oroantral communication risk. Dentist noticed bone exposure 2 months before the evaluation at Stomatology Clinics. Patient reported tooth extraction in the same region of bone exposure 2 years before and had no tooth or implants at the time of attendance to Stomatology Clinics. He also was advised to not use oral prosthesis during the period of MRONJ treatment, due to the risk of traumatizing oral mucosa. There was no other noteworthy oral alteration.

Conservative treatment was initiated with Clindamycin 600 mg/day, oral hygiene guidance, and topical application of chlorhexidine gluconate gel 0.12% at bone exposure on a daily basis. Although patient has been assisted biweekly in some special situations (mainly due to health issues), most of times he was followed up weekly and undertaken to superficial bone debridement, PDT, and LLLT application for 12 months, until clinical healing of bone exposure (Figure 1(c)), accounting a total of 37 sessions.

PDT, an effective therapy in reducing pathogens, consisted of staining the bone exposure with methylene blue 0.01% photosensitizer, waiting for 3 minutes before irradiation time, and applying red spectrum (*λ*660 nm) diode low-level laser (100 mW, 4 points of 4 J, 40 s/point, 142 J/cm^2^). In turn, intraoral LLLT was performed locally at alveolar ridge of maxilla with infrared spectrum (*λ*808 nm) diode low-level laser (100 mW, 12 points of 4 J, 40 s/point, 142 J/cm^2^), aiming at biomodulation of inflammation. Laser device used was THERAPY XT (DMC, São Carlos, SP, Brazil).

Patient was included in a follow-up regimen twice a month during 6 months after wound healing. He undertook infrared spectrum (*λ*808 nm) LLLT (100 mW, 12 points of 4 J, 40 s/point, 142 J/cm^2^) to prevent recurrence of MRONJ, which was not observed.

## 3. Discussion

According to the Position Paper on MRONJ of American Association of Oral and Maxillofacial Surgeons (AAOMS) 2014 Update [7], this case report was diagnosed at stage 2, which is defined as exposed necrotic bone or fistula that probes to bone associated with infection and/or suppuration.

Treatment strategies recommended by AAOMS should be symptomatic therapy with oral antibiotics, chlorhexidine gluconate 0.12% mouth rinses, pain relief, conservative debridement of bone sequestrum, and infection control. Beside these approaches, LLLT and PDT were applied as adjuvant treatment.

The management of MRONJ with LLLT and PDT was based on properties of these therapies and supported by metabolism-activating effects on bone and mucosa, both very well documented in literature.

Several authors have evaluated the biostimulatory effects of LLLT performed through different wavelengths on the trophism of bone and mucosa, both* in vitro* and* in vivo*. Reported phenomena include faster wound healing, increased fibroblast and chondroblasts proliferation, collagen synthesis, stimulation of osteogenesis, bone cells differentiation and bone repair mechanisms, increased blood flow, stimulation of endothelial cells proliferation, and analgesia [9–13]. Notwithstanding, PDT uses its light indirectly to trigger photosensitive dyes to produce bactericidal molecules (singlet oxygen), promoting an antimicrobial effect, thus killing potential pathogens. Indeed, current data indicates that PDT might be a useful therapy to treat oral infections [8, 9].

In this case report, it was evidenced that patient presented clinical improvement by treating MRONJ with LLLT and PDT. There was fistula remission, absence of bone necrosis, control of infection and/or suppuration, pain relief, and total repair of the oral mucosa through these adjuvant therapies. Once the lesion was no longer communicating with oral cavity also the maintenance of a preexisting infection and the risk of a new infectious conditions ceased to exist.

Vescovi et al. [14] tested the hypothesis that the combination of antibiotic therapy and LLLT could be effective in preventing MRONJ after tooth extraction in patients on BP therapy. Their result was out to meet ours, revealing minimal bone exposure. Corroborating to our findings, Altay et al. [15] stated that using LLLT not only promotes biostimulating properties, analgesia, and wound healing but also optimizes clinical evolution and treatment time when compared to conventional management. Latifyan et al. [16] further pointed out that this adjuvant treatment is not associated with any known side effects.

Petelin et al. [17] reported positive results in reduction of pathogens in periodontal pockets using PDT. Rosa et al. [18] showed the effectiveness of PDT* in vitro* at inactivating* S. aureus* biofilms in compact and cancellous bone specimens. Qiao et al. [19] adduced that PDT exhibited no cytotoxicity to human periodontal ligament cells and human gingival fibroblasts* in vitro*. Instead, it stimulated proliferation, attachment, and collagen synthesis of human periodontal ligament cells and human gingival fibroblasts. In this way, PDT appears to be a safe antimicrobial treatment that spares normal tissues from damaging effects and its use is justified on MRONJ, corroborating to our case report findings.

According to the results obtained in this case report study, it was found that PDT applied directly to exposed bone with suppuration can bring beneficial effects to control the infected MRONJ lesion. Furthermore, it was observed that LLLT promoted total repair of oral mucosa. Therefore, we can state that both were essential in approach and in success of disease control, reinforcing the importance of its applicability and indication. Although LLLT and PDT seem to be useful approaches in the management of MRONJ, more studies are necessary to elucidate the real benefits that these therapies can propose.

## 4. Conclusions

The findings of this case report study suggest that both LLLT and PDT brought important benefits to patient, assisting in clinical management of the MRONJ.

The proposed new therapeutic approach led to decreased stage of MRONJ lesion, acting as an adjuvant treatment within a set of clinical maneuvers, bringing beneficial effects to control the disease, and providing improved patient quality of life.

Based on results it is recommended to use LLLT and PDT as adjuvant treatment of MRONJ. It is also suggested that further researches be conducted to obtain more relevant data to the action of these therapies in the management of MRONJ lesions.
